# Draft Genome Sequence of Actinoplanes utahensis NRRL 12052, a Microorganism Involved in Industrial Production of Pharmaceutical Intermediates

**DOI:** 10.1128/genomeA.01411-14

**Published:** 2015-01-08

**Authors:** Rodrigo Velasco-Bucheli, Carlos del Cerro, Daniel Hormigo, Carmen Acebal, Miguel Arroyo, José L. García, Isabel de la Mata

**Affiliations:** a Department of Biochemistry and Molecular Biology I, Facultad de Biología, Universidad Complutense de Madrid, Madrid, Spain; b Department of Environmental Biology, Centro de Investigaciones Biológicas, Consejo Superior de Investigaciones Científicas, Madrid, Spain

## Abstract

Here, we describe the draft genome sequence of Actinoplanes utahensis NRRL 12052, a filamentous bacterium that encodes an aculeacin A acylase and a putative *N*-acyl-homoserine lactone acylase of biotechnological interest. Moreover, several nonribosomal peptide synthase (NRPS) and polyketide synthase (PKS) clusters and antibiotic resistance genes have been identified.

## GENOME ANNOUNCEMENT

Actinoplanes utahensis NRRL 12052 is a Gram-positive filamentous bacterium able to hydrolyze aliphatic acyl-side chains of many antimicrobials, such as penicillins (1), lipopeptides (2–6), glycopeptides (7, 8), and capsaicin (9). Its draft genome was obtained at the Fundación Parque Científico de Madrid (Spain) from a shotgun library constructed and sequenced using Titanium 454 GS-FLX instrument (Roche Diagnostics, Banford, CT) according to the manufacturer, except that the emulsion employed in the live amplification mix contained an emulsion PCR (emPCR) additive instead of water. The genome comprises 9.5 Mb with 71.2% G+C content, obtaining 396 large contigs from 2.1 × 10^6^ reads by Newbler 2.5.3, which were reduced to 141 contigs by manual assembly (39.4-fold coverage). Seventy-seven RNA genes (6 rRNA and 71 tRNA) and 8,744 coding sequences (CDSs) were detected by RAST (10), 730 assigned with putative functions and 3,010 hypothetical proteins, including a putative *N*-acyl-homoserine lactone acylase (AHLA) and the aculeacin A acylase (AAC) (1, 6), which play critical roles in the generation of building blocks to synthesize therapeutic antimicrobials. However, the physiological roles remain unknown. According to antiSMASH (11), this genome contains 24 clusters, some of them associated with nonribosomal peptide synthases (NRPSs), polyketide synthases (PKSs), and bacteriocin, among others. The AAC-encoding (*aac*) gene is located within the third cluster, involved in the biosynthesis of an NRPS with the monomer prediction (gly-nrp-nrp-nrp) + (nrp-nrp). The putative AHLA-encoding (*ahl*) gene is located at 88 kb upstream within the fifth cluster which codifies for another NRPS with the monomer prediction (nrp) + (tyr–pro).

The *ahl* gene encodes a protein of 808 amino acids (aa), which shows modular organization including a predicted 33-aa signal peptide (12), an α-subunit of 20.9 kDa, and a β-subunit of 60.4 kDa, This enzyme presents the essential catalytic amino acid that is conserved in other acylases (13), suggesting that it is involved in a quorum-quenching mechanism.

A single *aac* gene was detected in this microorganism, suggesting that the membrane-associated echinocandin B deacylase (5, 14, 15), which only differs from the soluble AAC form (6, 16) by two additional N-terminal amino acids, is encoded by the same gene.

Furthermore, RAST detected 26 subsystems involved in the metabolism of carbohydrates, amino acids, and proteins, among others. Likewise, genes involved in the mechanisms of virulence, disease, and defense were detected, highlighting the presence of tetracycline, fluoroquinolones, and vancomycin resistance genes as well as 16 β-lactamases.

A phylogenetic tree by MEGA6 (17) was inferred from 16S rRNA (1520 bp), showing that A. purpeobrunneus IFO 14020 and A. derwentensis IFO 14935 are closely located. However, a very evolutionary difference to other bacteria was displayed when a deep comparative analysis with its whole genome was performed by JSpecies (18).

### Nucleotide sequence accession numbers.

This whole-genome shotgun project has been deposited at GenBank under accession no. JRTT00000000. The version described in this paper is the first version, JRTT01000000.
